# A new mutation in the gene encoding mitochondrial seryl-tRNA synthetase as a cause of HUPRA syndrome

**DOI:** 10.1186/1471-2369-14-195

**Published:** 2013-09-13

**Authors:** Henry Rivera, Elena Martín-Hernández, Aitor Delmiro, María Teresa García-Silva, Pilar Quijada-Fraile, Rafael Muley, Joaquín Arenas, Miguel A Martín, Francisco Martínez-Azorín

**Affiliations:** 1 Laboratorio de Enfermedades Mitocondriales. Instituto de Investigación Hospital 12 de Octubre (i + 12), 6º Planta, Bloque E, Avda. Córdoba s/n, Madrid E-28041, Spain; 2 Centro de Investigación Biomédica en Red de Enfermedades Raras (CIBERER), U723, Madrid E- 28041, Spain; 3 Unidad Pediátrica de Enfermedades Raras, Hospital 12 de Octubre, Madrid E-28041, Spain; 4 Unidad Pediátrica de Nefrología, Hospital 12 de Octubre, Madrid E-28041, Spain

**Keywords:** Mitochondrial DNA, Mitochondrial disease, HUPRA syndrome, *SARS2*, Mitochondrial respiratory chain

## Abstract

**Background:**

HUPRA syndrome is a rare mitochondrial disease characterized by hyperuricemia, pulmonary hypertension, renal failure in infancy and alkalosis. This syndrome was previously described in three patients with a homozygous mutation c.1169A > G (p.D390G) in *SARS2*, encoding the mitochondrial seryl-tRNA synthetase.

**Case presentation:**

Here we report the clinical and genetic findings in a girl and her brother. Both patients were clinically diagnosed with the HUPRA syndrome. Analysis of the pedigree identified a new homozygous mutation c.1205G > A (p.R402H) in *SARS2* gene. This mutation is very rare in the population and it is located at the C-terminal globular domain of the homodimeric enzyme very close to p.D390G.

**Conclusion:**

Several data support that p.R402H mutation in *SARS2* is a new cause of HUPRA syndrome.

## Background

Mitochondria are cellular organelles that produce most of the energy that cells use for their functions and survival [1]. Their biogenesis and function is under the genetic control of mitochondrial DNA (mtDNA) and nuclear DNA. Therefore mitochondrial disorders can be originated from mutations in either of the genomes [2]. These disorders represent one of the most common groups of inborn errors of metabolism, with an estimated prevalence of 1 in 5000 births [3]. The kidneys are commonly affected in mitochondrial disease [4,5] because they are aerobic organs with high energy requirements [6].

We report the case of a girl and her brother who presented multiorgan disease, including hyperuricemia, pulmonary hypertension, renal failure, and alkalosis (HUPRA syndrome) (OMIM #613845) [7]. The sequencing of *SARS2* gene identified a new homozygous mutation (c.1205G > A p.R402H) as a potential cause of the syndrome.

## Case presentation

### Patient II-1

The patient was a girl born at 37 weeks of gestation of an uneventful pregnancy and delivery. She was the first child of non-consanguineous Spanish parents. At 15 months she was referred to our department because of refractory anemia since the age of 5 months, progressive renal failure with hyperuricemia since the age of 11 months, and metabolic hypochloremic alkalosis despite renal failure. Physical examination was normal except for pallor and mild hypotonia with motor delay. Her weight was 8.250 kg (percentile 11.5 using WHO reference), her height 76.0 cm (percentile 30.8) and her cranial perimeter 46 cm (percentile 61.8). Blood test revealed a normocytic normochromic anemia (Hb 8.4 g/dL (>12), VCM 75 fL (70–115), HCM 27 pg (23–35), with normal leucocytes, thrombocytes and reticulocytes), metabolic hypochloremic alkalosis (pH 7.51 (7.35-7.45), pCO_2_ 35 mmHg (27–40), HCO_3_^-^ 29.3 mmol/L (16–24), Cl^-^ 85 mmol/L (95–106)), renal failure with disproportionately higher urea than creatinine (creatinine 1.01 mg/dL (0.35–0.5), urea, 159 mg/dL (20–48), estimated creatinine clearance of 42 mL/min/1.73 m2 (>60)) and hyperuricemia (uric acid 11 mg/dL (2.2–7)). Urinalysis displayed a specific gravity of 1.015, pH 5.0, absence of proteinuria and normal cellular sediment. Abdominal ultrasound showed normal kidneys. Her bone marrow aspiration was normal and she had neither ringed sideroblasts nor vacuolization of hematopoietic precursors. Renal and muscle biopsies were performed at the age of 24 months. Renal histology showed normal glomeruli, interstitial fibrosis and tubular atrophy with vacuoles in proximal tubular epithelial cells. Electron microscopy revealed enlarged mitochondria in the cytoplasm of epithelial cells of the proximal tubules. Muscle biopsy demonstrated variations in fiber size, consistent with type 2 fiber atrophy. Cytochrome C oxidase (COX) and succinate dehydrogenase (SDH) histochemistry were normal. Electron microscopy study of the muscle was normal. Enzymatic activities of mitochondrial respiratory chain in skeletal muscle were normal (Table 1). Deletions of mtDNA were excluded in muscle sample. In additional studies the pancreatic function and the metabolic work up lactic acid, pyruvic acid, amino acids and acylcarnitines were also normal.

**Table 1:** Mitochondrial respiratory chain activities in patients with HUPRA syndrome

	**Skeletal muscle**			**Fibroblasts**	
**Enzyme**	**II-1**	**II-2**	**C (mean ± SD)**^**c**^	**II-2**	**C (mean ± SD)**^**d**^
**Citrate synthase (CS)**^**a**^	455	298	550 ± 350	76	87 ± 13
**Complex I**^**b**^	14.8	19.5	15.9 ± 5.9	**13.8**	19.5 ± 3.9
**Complex II**^**b**^	14.0	11.0	12.5 ± 8.0	41.8	39.7 ± 2.5
**Complex III**^**b**^	73.7	34.8	68.8 ± 37.8	63.6	63.1 ± 6.5
**Complex IV**^**b**^	53.6	43.8	66.5 ± 36.5	**74.8**	86.3 ± 4.2

^a^In SA (SA is specific activity: nmol × min^-1^ × mg protein^-1^).

^b^In (SA of Complex / SA of Citrate Synthase) × 100.

^c^Skeletal muscle from controls: N = 14.

^d^Fibroblasts from controls: N = 6.

Bold data indicate that they are below the normal range.

In the following months the child required repeated blood transfusions in spite of having received erythropoietin treatment. She presented failure to thrive, hypertension and progressive renal failure. Her overall clinical condition gradually deteriorated and she died at 26 months of multiorgan failure. In the necropsy the only relevant finding was hypertrophic cardiomyopathy.

### Patient II-2

**Figure 1:**
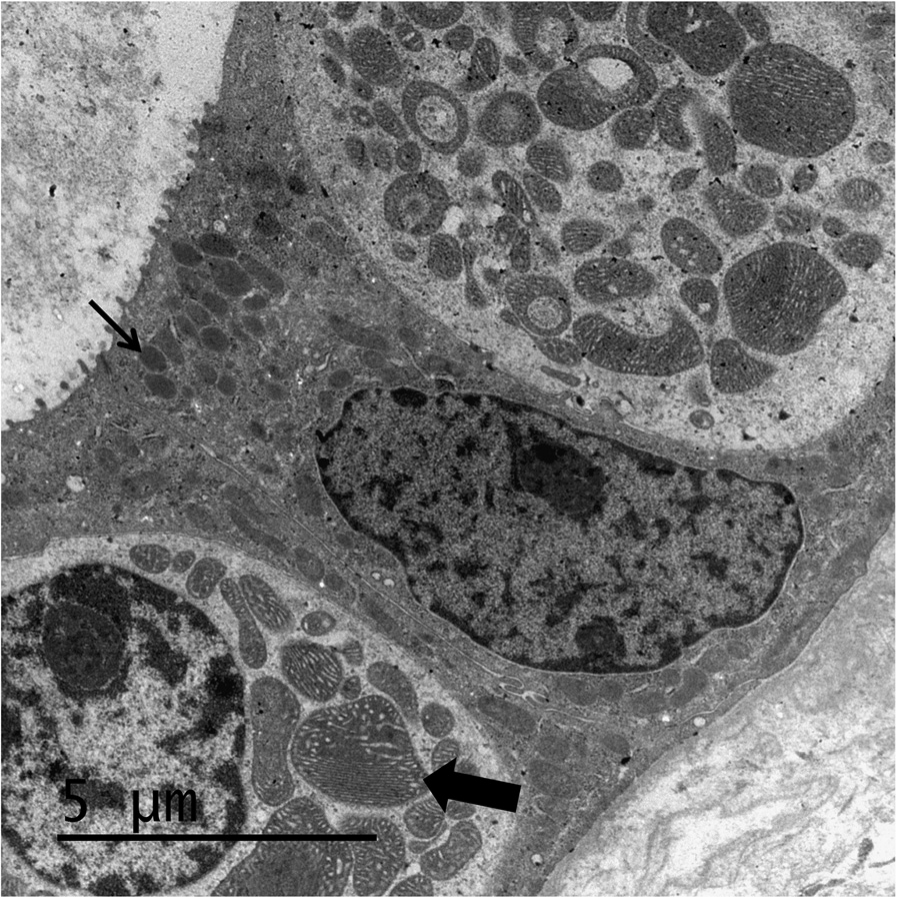
**Electron micrograph of a kidney tubule.** Abnormal enlarged mitochondria in a portion of the tubular epithelial cells obtained from patient II-2. Normal mitochondrion is marked with a thin arrow and a giant mitochondrion with a wide arrow.

The patient was the younger brother of patient II-1. He was born at 36 weeks of gestational age and had an uneventful neonatal period. At 2 months a normocytic normochromic anemia was found with levels of Hb of 7.6 g/dL, VCM 83 fL, HCM 28 pg, which required repeated blood transfusions since 9 months. Bone marrow was normal even on electron microscopy. At 3 months, a hyperuricemia was found with uric acid levels of 6.6 mg/dL which increased to 9.4 mg/dL at the age of 9 months. The fractional excretion of uric acid (FeUA) was constantly low with values of 2-3% (>7%). As a result of failure to thrive since the age of 7 months, continuous nasogastric night feeding and nutritional supplements were administered at the age of 12 months. At 7 months he had hyponatremia with an increased fractional excretion of sodium, and elevated creatinine (0.96 mg/dL) during a respiratory infection. Similar to his older sister, urea levels were always disproportionately higher than creatinine values and hypochloremic alkalosis was present despite renal insufficiency. Serum magnesium levels were 1.7 mg/dL (1.5-2.3). Abdominal ultrasound showed normal kidneys. Moreover, lactic acid, amino acids and acylcarnitines in plasma, and organic acids in urine were normal. At 15 months he had normal social and cognitive development with a mild motor delay, his weight was 7.860 kg (percentile 1.8 using WHO reference), his height 71.5 cm (percentile 0.1) and his cranial perimeter 44 cm (percentile 1.8). Muscle biopsy was performed and histology, histochemistry and enzymatic studies of respiratory chain were normal (Table 1). Mitochondrial DNA deletions, depletion and frequent mutations were excluded. Other nuclear genes were sequenced with normal results (*HNF1, UMOD, CoQ2* and *PDSS2*). A catheter was placed and ambulatory peritoneal dialysis was started due to creatinine values of 6.3 mg/dL. A renal biopsy was performed at 17 months showing interstitial fibrosis and unspecific tubular damage with normal glomeruli and vascular elements by optic microscopy. Giant and abnormal mitochondria were observed by electron microscopy (Figure 1). Enzyme analysis of respiratory chain complexes in cultured fibroblasts from a skin biopsy at 17 months revealed mild reduced activity of complexes I and IV (Table 1).

At 16 months, severe pulmonary hypertension and right ventricular hypertrophy were diagnosed by echocardiography. In the hemodynamic study, pressures of pulmonary arteries were at the same level as systemic pressures which improved with oxygen and nitric acid administration. Treatment with sildenafil and bosentan showed a partial response. At 21 months he was hospitalized for pneumonia and died of pulmonary hemorrhage, refractory pulmonary hypertension and cardiac failure.

### Biochemical studies

Both patients with HUPRA syndrome were studied for OXPHOS deficiencies in skeletal muscle homogenate [8], and the patient II-2 also in skin fibroblasts [9]. These two methodologies are slightly different being the methodology used in fibroblasts more sensitive and reproducible. The activities of respiratory chain complexes in skeletal muscle homogenate of both patients were normal (Table 1). However, the patient II-2 showed a mild deficiency of complex I and complex IV activities in cultured skin fibroblasts (Table 1), with residual activities being 71% and 87% of the mean control values (less than one standard deviation (*1SD*) away from the *mean* value).

### *SARS2* sequencing

**Figure 2:**
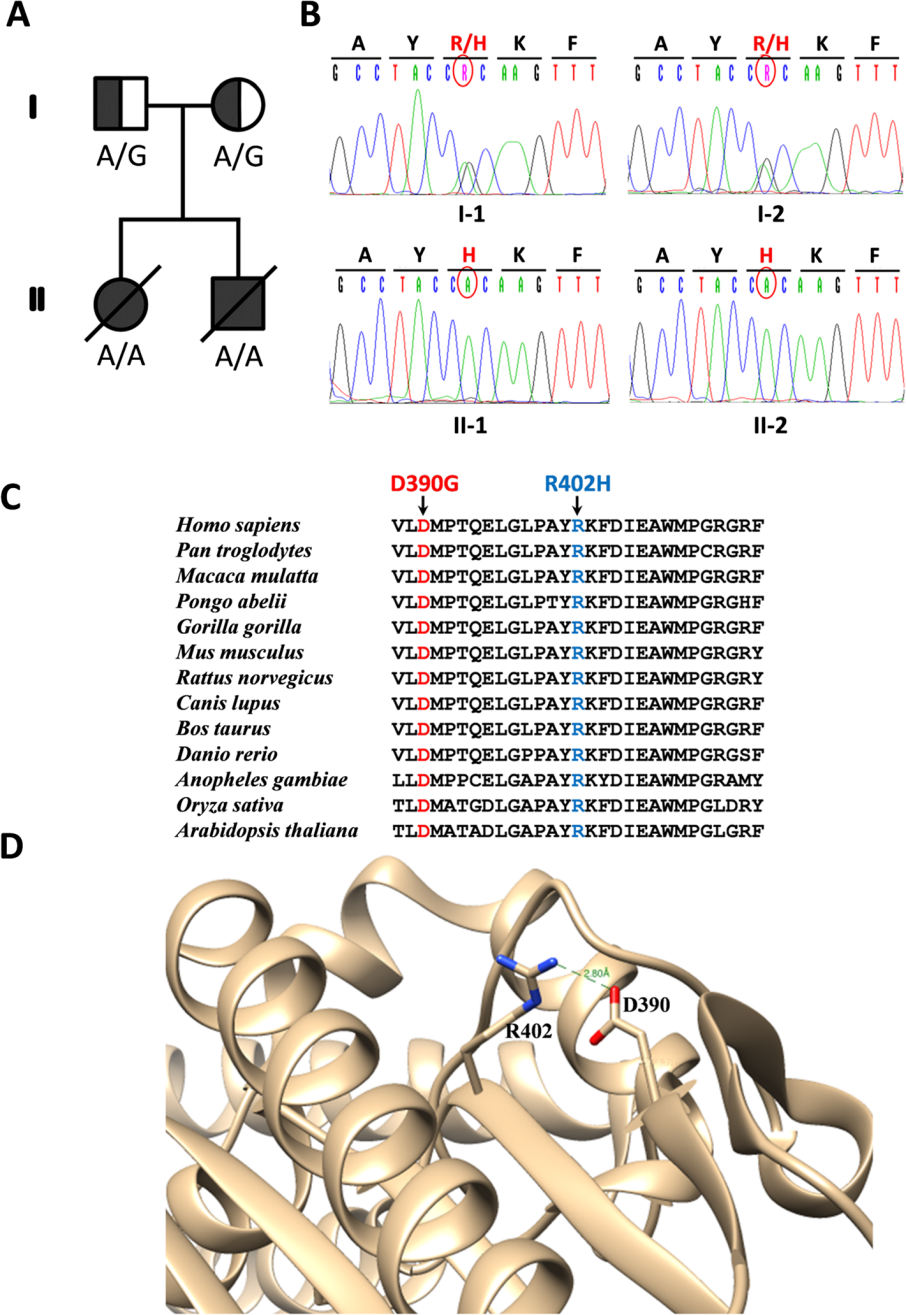
**Validation and segregation of c.1205G > A (p.R402H) mutation in *SARS2*. (A)** Family pedigree. **(B)** Electropherograms showing Sanger sequence validation of the *SARS2* c.1205G > A (p.R402H) mutation. **(C)** Multiple sequence alignment of SARS2 protein region surrounding the novel R402H mutation (blue) in various species. The position of D390G mutation is also indicated (red). **(D)** Spatial localization of R402 (blue) and D390 (red) is shown in the model of 3D structure of SARS2 protein (PDB code 1WLE). R402 is mutated in patients II-1 and II-2 and D390 is mutated in previously described patients with HUPRA syndrome.

Direct DNA sequencing by Sanger method of all the exons and the adjacent exon-intron boundaries of *SARS2* gene [10] was carried out in patient II-2. One homozygous mutation was found (c.1205G > A) resulting in p.R402H. The same missense mutation in a homozygous fashion was detected in patient II-1. And as expected, the parents (I-1 and I-2,) presented the mutation in heterozygosity (Figure 2A and B). The aminoacid change affects a highly conserved residue (Figure 2C) and the scores for pathogenicity obtained by *in-silico* analysis were “damaging” for SIFT [11] and “probably damaging” for PolyPhen-2 [12] software.

## Conclusions

Renal involvement is frequently observed in mitochondrial diseases [4,5], and therefore mitochondrial dysfunction should be considered in the differential diagnosis for unexplained renal disease in infancy [7,13]. We have studied two related infants (sister (patient II-1) and brother (patient II-2)) who presented failure to thrive, anemia, metabolic hypochloremic alkalosis, pulmonary hypertension and progressive renal failure with hyperuricemia. They also showed interstitial fibrosis, tubular damage and abnormal mitochondria in kidney. Additionally, one of them also showed a mild deficiency of the mitochondrial respiratory chain in fibroblasts (Table 1). The patients were comprehensively investigated for common etiologies of anemia, uric acid metabolism and mitochondrial metabolism, including sequencing of several genes (*HNF1, UMOD, CoQ2, PDSS2* and partially the mtDNA) but no underlying cause was identified. The only reported patients with similar clinical characteristics (3 patients from 2 unrelated Palestinian families from the same village) were diagnosed with the HUPRA syndrome [7] and therefore the patients II-1 and II-2 were clinically diagnosed with this syndrome. The genetic basis of HUPRA syndrome in Palestinian patients was a homozygous mutation (c.1169A > G p.D390G) in the *SARS2* gene (RefSeq NG_031865.1) [10], which encodes mitochondrial seryl‒tRNA synthetase [7]. The patients II-1 and II-2, unlike the Palestinian patients, had a more severe anemia, a milder developmental delay, normal levels of blood lactate, and normal COX histochemistry and mitochondrial respiratory chain activities in muscle. However, normal respiratory chain activities and morphology in muscle does not rule out the diagnosis of mitochondrial dysfunction. Thus, similar situations have been described before where respiratory chain activities in fibroblasts showed deficiencies while respiratory chain activities and histochemical analysis in muscle were normal [14]. On the other hand, lactic acid elevation in blood is an important, although non-specific and non-sensitive, marker of mitochondrial disease. Many patients with mitochondrial diseases consistently have normal lactic acid levels, even it has been described pathogenic mutations in *DARS2* where elevated lactate was only observed in the affected tissue (white matter) and not in blood or cerebrospinal fluid [15]. Additionally, from a cohort of 113 pediatric patients with mitochondrial disease, a significant respiratory chain defect was found in 71% of the patients, focal absence of COX activity was found only in 13% of patients and elevation of plasma lactic acid in 60% of them [16]. Thus, reaching a diagnosis of mitochondrial disease in pediatric patients can be challenging due to it can be accompanied by normal muscle morphology, normal plasma lactate, normal mitochondrial enzymes in skeletal muscle, normal mtDNA mutation screening, and a non-classical clinical presentation.

The description of HUPRA syndrome and it genetic origin due to a mutation in *SARS2* gene [7] prompted us to sequence this gene in the patients II-1 and II-2. The sequencing identified a very rare missense mutation in homozygous (c.1205G > A) in both patients that changes a conserved arginine to histidine (p.R402H). The mutation is very rare because its allelic frequency is 0.000154 in the population (13006 alleles), and the frequency of homozygous is 0 (in 6503 chromosomes). It has been described only twice in heterozygous in the Exome Sequencing Project (ESP) (https://esp.gs.washington.edu/drupal/). Furthermore, the mutation was predicted to have deleterious effect in the protein by analysis with SIFT [11] and PolyPhen-2 [12] software. It is located in one of the most conserved region of SARS2 and the aminoacid is conserved from *Homo sapiens* to *Arabidopsis thaliana* (Figure 2C). The mutation segregates with the disease in this family (Figure 2A and B): the same mutation was detected in patients II-1 and II-2 with identical phenotype, and the parents (I-1 and I-2,) were carriers of the mutation.

SARS2 is a homodimeric enzyme whose active site C-terminal globular domain is built around an eight antiparallel β-sheet, encompassed by three helical bundles. The aminoacids D390 and R402 are positioned at the C-terminal globular domain, D390 is in the second and R402 in the third β-strands surrounding an α-helix and a β-turn [17]. In the three-dimensional structure of the protein, both residues are very close (2.8 Å) (Figure 2D) [18], which could explain why mutations in these residues give rise to similar clinical phenotypes.

Recently, mutations in genes encoding mitochondrial aminoacyl-tRNA synthetases (mtARSs) have emerged as a new cause of human disease, resulting in surprisingly tissue-specific phenotypes, although they are all expected to impair mitochondrial protein synthesis and thus affect the OXPHOS system. However, the molecular mechanisms behind the selective tissue involvement are not currently understood. Several possibilities have been pointed out to explain the tissue-specificity phenotype of mtARS mutation [19]: (i) the remaining residual activity of mtARS is sufficient to maintain mitochondrial translation in most cell types but not in the specific tissues; (ii) tissue-specific differences in mitochondrial chaperone activities may play a role in determining the stability of some mutants; (iii) tissue-specific levels of particular uncharged tRNAs and amino acids; and (iv) mtARS could also have additional functions that are indispensable in specific cell types or developmental stages.

The principal affected tissue in HUPRA syndrome by *SARS2* mutations is the kidney. Mutations in *SARS2* resulting in decreased aminoacylation of one tRNA isoacceptor (tRNA^Ser^_AGY_ or tRNA^Ser^_UCN_) by the serine amino acid are expected to adversely affect mitochondrial translation systems and lead to derangements in the synthesis of mitochondrial proteins and consequently in energy supply. Reduced energy production may account for impaired tubular function, which is known to be especially vulnerable to mitochondrial dysfunction since it has very high energy requirements [6].

In conclusion, there are several findings that support the pathogenic role of R402H change: (i) the mutation is very rare in the population; (ii) the substitution is predicted to have deleterious effect in the protein; (iii) the aminoacid residue is highly conserved (Figure 2C); (iv) the mutation segregates with the disease (Figure 2A); (v) the patient fibroblasts have a combined deficiency in complexes of the mitochondrial respiratory chain (Table 1) as expected for defects in a gene involved in mitochondrial protein synthesis; and (vi) the mutation is located close to the only described mutation in *SARS2* associated with HUPRA syndrome (Figure 2D) [7]. Finally, we consider that the *SARS2* gene must be the first line of investigation when the HUPRA phenotype is present.

## Consent

Informed written consent was obtained from the patient’s parents, and the Ethic Committee of the Instituto de Investigación Hospital 12 de Octubre (i + 12) approved the study.

## Abbreviations

HUPRA: Hyperuricemia, pulmonary hypertension, renal failure, and alkalosis; SARS2: Mitochondrial seryl-tRNA synthetase; mtDNA: Mitochondrial DNA; ESP: Exome Sequencing Project; WHO: World Health Organization

## Competing interests

The authors have declared that no competing interests exist.

## Authors’ contributions

HR carried out molecular genetic studies and helped to draft the manuscript. EMH, MTGS, PQF, RM and JA participated in clinical evaluation. AD carried out enzymatic and *in silico* studies. MAM helped to draft the manuscript. FMA conceived and designed the experiments, analyzed the data and drafted the manuscript. All authors read and approved the final manuscript.

## Pre-publication history

The pre-publication history for this paper can be accessed here:

http://www.biomedcentral.com/1471-2369/14/195/prepub
